# Evolution of lung adenocarcinoma from preneoplasia to invasive adenocarcinoma

**DOI:** 10.1002/cam4.5393

**Published:** 2022-11-03

**Authors:** Jianfei Zhu, Wenchen Wang, Yanlu Xiong, Shuonan Xu, Jiankuan Chen, Miaomiao Wen, Yabo Zhao, Jie Lei, Tao Jiang

**Affiliations:** ^1^ Department of Thoracic Surgery Tangdu Hospital, Fourth Military Medical University Xi'an China; ^2^ Department of Thoracic Surgery Shaanxi Provincial People's Hospital Xi'an China

**Keywords:** adenocarcinoma in situ, drive gene, invasive adenocarcinoma, prognosis

## Abstract

**Objective:**

Mutations in driver genes contribute to the development and progression of lung adenocarcinoma (LUAD). However, in the dynamic evolutionary process from adenocarcinoma in situ (AIS) to minimally invasive adenocarcinoma (MIA) and eventually to invasive adenocarcinoma (IAC), the role of driver genes is currently unclear. This study aimed to analyse the role of driver gene status in the progression of LUAD from preneoplasia to IAC.

**Methods:**

Patients with LUAD who underwent surgery in our centre from March 2015 to December 2019 were retrospectively analysed, and LUAD patients with tumour sizes ≤3.0 cm and pN0 were included in the final analysis. The mutation status of common driver genes, including EGFR, ALK and ROS1, was detected. According to the pathological characteristics, the patients were divided into three stages: AIS, MIA and IAC. We analysed the distribution of driver gene mutation frequencies across three stages of LUAD. In addition, we performed univariate and multivariate analyses of IAC patients to screen for relevant variables (driver genes and clinicopathological features) affecting their prognosis.

**Results:**

Ultimately, 759 patients with LUAD were enrolled, including 135, 130, and 494 cases of AIS, MIA, and IAC, respectively. EGFR mutations were identified in 359 (61.8%) patients, and with the transition from AIS to MIA, the frequency of EGFR mutations increased from 33.3% to 50.8%, *p* = 0.004, whereas the frequency of EGFR mutations was comparable for MIA and IAC (50.8% vs. 50.2%, *p* = 0.922). Moreover, ALK and ROS1 gene fusions were identified in 17 cases (2.2%) and 2 cases (3.0‰) respectively. For AIS, neither ALK gene nor ROS1 gene fusions were observed. When the tumour progressed to MIA, the ALK fusion frequency was 2.3% (3/130), which was basically consistent with the ALK fusion frequency of 2.8% in IAC, *p* = 0.143. For IAC, fusions of ROS1 fell into this category. In addition, we found that 40 patients (5.3%) developed metastasis/recurrence, and 14 patients (1.8%) died of cancer‐specific related diseases. Notably, for AIS, there were no recurrences and no deaths, and for MIA, only 1 patient died with LUAD. Finally, survival analysis was performed in patients with stage IA invasive adenocarcinoma, and EGFR‐mutant patients showed better DFS than EGFR‐wild‐type patients (*p* = 0.036). Conversely, patients with ALK fusions showed worse DFS than those with ALK wild‐type (*p* = 0.004), and the same results were found in OS analysis.

**Conclusions:**

The accumulation of EGFR driver gene mutation frequencies mediates the progression of LUAD from AIS to MIA. When the tumour progresses to stage IA invasive adenocarcinoma, multivariate analysis based on driver gene status can be used as a pivotal prognostic factor.

## Graphical abstract

The role of driver gene mutation in the invasion process of lung adenocarcinoma.

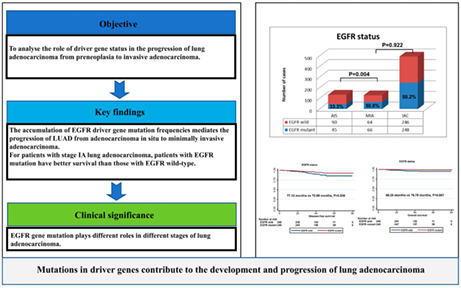

## 1 INTRODUCTION

The National Lung Screening Trial (NLST) confirmed that low‐dose spiral computer tomography (CT) increases the lung cancer detection rate by 17.3% and reduces lung cancer mortality by 20% (95% CI 6.8 to 26.7; *p* = 0.004).^1^ As low‐dose spiral CT is widely used in health checkups, an increasing number of lung nodules are screened. According to radiological characteristics, lung nodules can be divided into pure ground‐glass nodules (pGGNs), subsolid nodules (SSNs) and solid nodules (SNs).^2^, ^3^ For these malignant lung nodules, the histopathology type is mainly lung adenocarcinoma (LUAD), ranging from adenocarcinoma in situ (AIS) to minimally invasive adenocarcinoma (MIA) and invasive adenocarcinoma (IAC), showing a gradual progression trend.^4^, ^5^ Indeed, the dynamic evolution of AIS‐MIA‐IAC is the focus of current research, but the results are not satisfactory.

Recently, the fifth edition of the WHO Classification of Thoracic Tumours removed AIS from the LUAD list, classifying it as a precursor gland lesion, while MIA remains classified as LUAD, suggesting that AIS may be heterogeneous to MIA.^6^ Data from long‐term survival may confirm that both AIS and MIA have an excellent prognosis of 100% 5‐year survival but that when the tumour progresses to the IAC stage, the prognosis is significantly worse than that of AIS and MIA.^7^, ^8^ Previous studies^9^, ^10^ have shown that the driver gene mutation status as a predictor of the efficacy of TKIs may play a role in the progression of LUAD from AIS to IAC, but conclusive evidence is still lacking.

To address our knowledge gap in the dynamic evolution of AIS‐MIA‐IAC, we propose that driver gene mutation status mediates the progression of LUAD from precancer (AIS) to IAC. We consecutively selected 759 patients with LUAD after strict screening and found that an increase in EGFR mutation frequency may be involved in the progression from AIS to MIA. When LUAD progresses to the invasive adenocarcinoma stage, in some specific subtypes, EGFR mutation may be a better prognostic factor.

## 2 MATERIALS AND METHODS

### 2.1 Study design

From March 2015 to December 2019, we consecutively enrolled patients with LUAD who underwent radical resection in our hospital. The inclusion criteria were as follows: (1) histological diagnosis of LUAD; (2) sufficient specimens for driver gene testing; (3) R0 resection; (4) tumour diameter of ≤3.0 cm; (5) solitary pulmonary nodule; (6) pN0 stage; (7) without neoadjuvant therapy and adjuvant therapy, and (8) no other distant organ metastasis. All patients meeting the following conditions were excluded from this study: (1) atypical hyperplasia (AAH); (2) mucinous adenocarcinoma; (3) previous history of other malignant tumours; (4) perioperative death; and (5) expected survival less than 3 months. This work has been reported in line with the STROCSS criteria.^11^ The clinical trial registration number for this study is ChiCTR2200059416.

### 2.2 Histopathological and radiological diagnosis

Postoperative pathological diagnosis was based on the lung cancer classification system by the WHO (5th edition) by three independent experienced pathologists. (1) AIS is histologically defined as a small localised adenocarcinoma (≤3.0 cm) with cancer cells growing strictly along preexisting alveolar structures in a lepidic growth pattern, lacking spread through air spaces (STAS), lymphovascular invasion (LVI), or pleural invasion and without acinar, papillary, solid, or micropapillary lesions; (2) MIA is defined as a solitary and discrete small adenocarcinoma (≤3.0 cm) with predominantly mural growth, and the largest diameter of invasion in any section is ≤5 mm. (3) IAC refers to adenoid differentiation and expression of alveolar malignant markers, invading into the interstitium of myofibroblasts for more than 5 mm in acinar, papillary, solid, micropapillary and other growth patterns, or cancer cells presenting as LVI, STAS, and pleura invasion, or tumour necrosis.^6^ IAC can be divided into lepidic pattern‐predominant adenocarcinoma (LPA), acinar pattern‐predominant adenocarcinoma (APA), solid pattern‐predominant adenocarcinoma (SPA), papillary pattern‐predominant adenocarcinoma (PPA) and micropapillary pattern‐predominant adenocarcinoma (MPA) according to the predominant subtype.

The radiological classification of tumour was determined on the basis of the thin‐section CT scan.^12^ The window level for lung nodule assessment is −500 to −700 HU, and the width of lung window is set as 1500–2000 HU. Consolidation tumour ratio (CTR) is defined as the ratio between the diameter of the largest solid component and the largest tumour diameter on CT. All nodules were identified as three types: pGGN: homogeneous ground glass nodules without solid components; SSN: the tumour contains solid components, but CTR < 0.75; SN: 0.75 ≤ CTR ≤ 1.

### 2.3 Driven gene analysis

DNA and RNA were extracted from fresh frozen tissue after surgery for subsequent EGFR mutation, ALK and ROS1 gene fusion detection (Xiamen Eder Biological Co., Ltd.). Using the ARMS method, the extracted DNA samples were configured with a qRT‐PCR system for mutation detection, covering 29 EGFR mutation hotspots from exons 18 to 21. RNA samples were reverse transcribed to obtain cDNA, which was then used for qRT PCR detection of ALK and ROS1 fusion genes. The testing process and the definition of positive results were described in detail in our previous study.^13^, ^14^, ^15^

### 2.4 Follow‐up

According to the clinical diagnosis and treatment standards, all patients are followed up strictly after surgery, and the first and third 6‐month follow‐ups within the first 2 years after surgery include basic information, physical examinations, lung cancer markers and chest CT scans. A comprehensive examination should be performed annually after surgery, and in addition to the above information, abdominal CT, ECT, whole‐brain CT/MRI, or PET/CT should be added. If the tumour recurs, additional information, including the site of recurrence and treatment, is needed. In this study, the last follow‐up time was December 31, 2021, and all patients underwent a final assessment of survival status.

### 2.5 Statistical analysis

The chi‐square test and Fisher's exact test were used to compare the correlation between different driver gene statuses and clinicopathological characteristics. The survival curves of disease‐free survival (DFS) and overall survival (OS) were drawn using the Kaplan–Meier method, and the log‐rank test was used to compare the differences between groups. Significant variables in univariate analysis were incorporated into Cox regression models for multivariate analysis. All tests were two‐tailed, and *p* values <0.05 were identified as statistically significant. All statistics were performed in SPSS (version 22.0) and STATA (version 14.0).

## 3 RESULTS

### 3.1 Clinicopathological characteristics of the included patients

**FIGURE 1:**
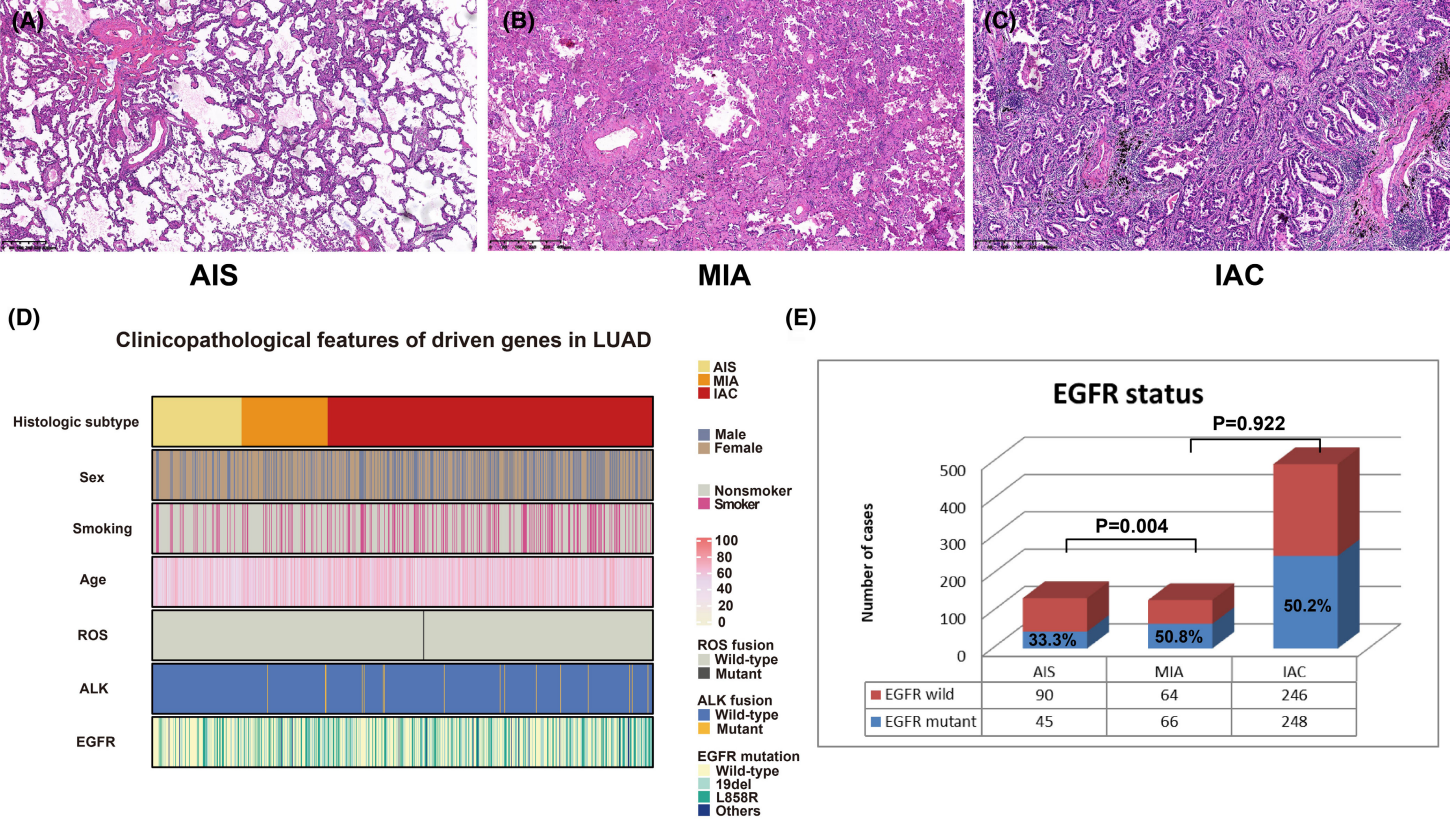
Clinicopathological characteristics and driver gene mutation status of the enrolled patients. (A–C) Typical pathological features of AIS, MIA and IAC. (D) Distribution of clinicopathological features and driver gene status among 759 patients. (E) EGFR mutation frequency in three stages of AIS‐MIA‐IAC.

A total of 759 patients were enrolled, including 135, 130, and 494 cases of AIS, MIA, and IAC, respectively (Figure 1A–C). A total of 471 (62.1%) were female, and 554 (73.0%) were nonsmokers. The median age was 58.0 years (range: 28.0–82.0 years). According to radiological subtype, 10 (27.6%), 182 (24.0%) and 367 (48.4%) patients were classified as pGGN, SNN and SN, respectively. Most tumours had diameters between 1.0 and 2.0, accounting for 55.6% (442/779). Lobectomy and sublobar resection were performed in 517 (68.1%) and 242 (31.9%) patients, respectively. The clinicopathological characteristics of all these cases are presented in Table 1.

**TABLE 1:** Differences among clinicopathological features of three stages of AIS, MIA, and IAC in LUAD (*N* = 759)

Variable	Pathological subtype				
	*N*	AIS	MIA	IAC	*p* value
Sex					<0.001
Male	288	34	40	214	
Female	471	101	90	280	
Age					<0.001
<58 years	353	85	62	206	
≥58 years	406	50	68	288	
Smoking status					0.002
Smoker	205	24	27	154	
Nonsmoker	554	111	103	340	
Radiological subtype					<0.001
pGGN	210	81	52	77	
SSN	182	29	53	100	
SN	367	25	25	317	
Tumour size					<0.001
≤1.0 cm	175	76	37	62	
>1.0 cm, ≤2.0 cm	422	51	79	292	
>2.0 cm, ≤3.0 cm	162	8	14	140	
EGFR mutation status					0.002
Mutant	359	45	66	248	
Wild	400	90	64	246	
ALK mutation status					0.143
Mutant	17	0	3	14	
Wild	742	135	127	480	
ROS1 mutation status					0.584
Mutant	2	0	0	2	
Wild	757	135	130	492	
Surgical approach					<0.001
Lobectomy	517	46	63	408	
Sub‐lobectomy	242	89	67	86	

### 3.2 Distribution of driver gene status in three stages of LUAD from AIS to IAC

We analysed the correlation between the three pathological stages (AIS, MIA, and IAC) and clinicopathological features (Table 1; Figure 1D). AIS was mainly found in female patients, young people and nonsmokers, accounting for 74.8%, 63.0% and 82.2% of cases, respectively. Likewise, patients with AIS were most likely to have pGGN (60.0%) and tumour diameters of ≤1.0 cm (56.3%). Moreover, 89 (65.9%) patients with AIS underwent sublobar resection.

Additionally, the EGFR mutation status was analysed, and EGFR mutations were identified in 359 (47.3%) patients. EGFR mutations involved deletions in exon 19; L858R in exon 21 was the most common mutation type, observed in 185 (51.5%) and 159 (44.3%) patients, respectively, and the remaining 15 patients had other uncommon mutation types (4.2%). Interestingly, with the transition from AIS to MIA, the frequency of EGFR mutations increased from 33.3% to 50.8%, *p* = 0.004, whereas the frequency of EGFR mutations was comparable for MIA and IAC (50.8% vs. 50.2%, *p* = 0.922), as shown in Figure 1E.

ALK and ROS1 gene fusions were identified in 17 patients (2.2%) and 2 patients (3.0‰), respectively. Notably, about 5.2% (4 cases) of patients were identified as coexistence of EGFR mutation and ALK fusion, including 1 patient of AIS and 3 patients of IAC. For AIS, we did not find ALK gene fusions. When the tumour progressed to MIA, the frequency of ALK fusion was 2.3% (3/130), which was basically consistent with the ALK fusion frequency of 2.8% in IAC, *p* = 0.143. For ROS1 fusion, only two patients with ROS1 fusion had IAC, *p* = 0.584.

### 3.3 Prognostic value of driver genes in stage IA invasive adenocarcinoma

The median follow‐up time for all patients was 42.57 months. By the last follow‐up time, 40 patients (5.3%) had developed metastasis/recurrence, and 14 patients (1.8%) had died of cancer‐specific related diseases. Notably, for AIS, there were no recurrences and no deaths, and for MIA, only 1 patient died with LUAD. Because AIS and MIA have excellent prognoses, here we will only analyse the prognostic value of driver genes in IAC. Moreover, only three patients of IAC were identified as coexistence of EGFR mutation and ALK fusion, so these patients were not excluded in survival analysis.

Of the 494 evaluated cases of stage IA invasive adenocarcinoma, the relationship between driver gene status and clinicopathological features is shown in Table 2. A total of 249 patients (50.4%) had EGFR mutations, 14 patients (2.8%) had ALK fusions, and 2 patients (4‰) had ROS1 fusions. As expected, EGFR mutations were frequently observed in women (*p* < 0.001), never smokers (*p* < 0.001) and LPA (*p* < 0.001).

**TABLE 2:** Correlation between driver gene status and clinicopathological features in stage IA invasive adenocarcinoma (*N* = 494)

Variable	*N*	EGFR	*p* value	ALK	*p* value	ROS1	*p* value
Sex			<0.001		0.413		1.000
Male	214	85		8		1	
Female	280	163		6		1	
Age			0.715		0.277		0.173
<58 years	206	101		8		2	
≥58 years	288	147		6		0	
Smoking status			<0.001		1.000		0.527
Smoker	154	57		4		1	
Nonsmoker	340	191		10		1	
Radiological subtype			0.179		0.172		0.541
pGGN	76	37		0		0	
SSN	101	59		2		1	
SN	317	152		12		1	
Predominant subtype			<0.001		0.167		0.532
LPA	217	120		2		0	
APA	192	106		7		2	
SPA	37	8		2		0	
PPA	28	7		2		0	
MPA	20	7		1		0	
pT stage			0.393		0.103		0.499
pT1a	62	29		0		0	
pT1b	292	142		12		2	
pT1c	140	77		2		0	

**FIGURE 2:**
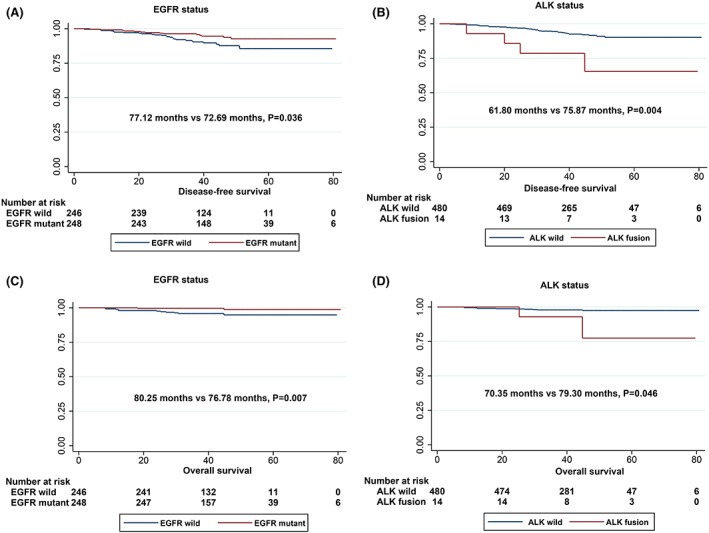
Survival curves of patients with stage IA invasive adenocarcinoma based on the driver gene mutation status. (A) DFS curves according to EGFR mutation status. (B) DFS curves according to ALK fusion status. (C) OS curves according to EGFR mutation status. (D) OS curves according to ALK fusion status.

**FIGURE 3:**
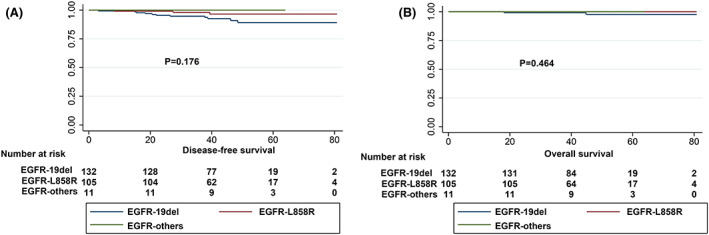
Survival curves stratified by EGFR mutation type. (A) DFS curves according to EGFR mutation type. (B) OS curves according to EGFR mutation type.

Next, we performed DFS and OS analysis of stage IA invasive adenocarcinoma, and in univariate analysis, sex (*p* = 0.007), smoking status (*p* = 0.034), radiological type (*p* = 0.001), and predominant subtype (*p* < 0.001) were significant variables affecting DFS in patients with stage IA invasive adenocarcinoma. Notably, EGFR‐mutant patients showed better DFS than EGFR‐wild‐type patients (*p* = 0.036), as shown in Figure 2A. Conversely, patients with ALK fusions showed worse DFS than those with wild‐type ALK (*p* = 0.004) (Table 3; Figure 2B). In multivariate analysis, radiological type (HR: 3.45, *p* = 0.021) and predominant subtype (HR: 0.36, *p* = 0.018) were independent predictive factors for DFS from stage IA invasive adenocarcinoma (Table 4). Moreover, in the OS analysis, patients with EGFR mutations had significantly longer OS than patients with wild‐type EGFR (*p* = 0.007), and patients with ALK fusion had shorter OS (*p* = 0.046) (Figure 2C,D). However, no meaningful variables were found in the multivariate analysis, as summarised in Tables 3 and 4. In the subanalysis, DFS and OS did not differ among the various EGFR mutation types (Figure 3A,B).

**TABLE 3:** Univariate analysis of DFS and OS in patients with stage IA invasive adenocarcinoma (*N* = 494)

Variable	DFS	95% CI	*p* value	OS	95% CI	*p* value
Sex			0.007			0.027
Male	73.06	70.26–75.87		77.71	75.84–79.57	
Female	77.36	75.57–79.14		80.12	79.35–80.89	
Age			0.865			0.997
<58 years	75.46	73.12–77.80		79.07	77.77–80.38	
≥58 years	75.26	73.04–77.48		78.90	77.58–80.21	
Smoking status			0.034			0.165
Smoker	72.85	69.48–76.21		77.73	75.52–79.94	
Nonsmoker	76.69	74.96–78.42		79.70	78.82–80.58	
Radiological type			0.001			0.034
pGGN	69.32	66.96–71.67		NA	NA	
SSN	60.08	59.27–60.90		NA	NA	
SN	73.60	71.37–75.83		NA	NA	
pT stage			0.459	NA	NA	0.330
pT1a	NA	NA		NA	NA	
pT1b	NA	NA		NA	NA	
pT1c	NA	NA		NA	NA	
Predominant subtype			<0.001			0.001
LPA	NA	NA		NA	NA	
APA	NA	NA		NA	NA	
SPA	NA	NA		NA	NA	
PPA	NA	NA		NA	NA	
MPA	NA	NA		NA	NA	
EGFR mutation status			0.036			0.007
Mutant	77.12	75.23–79.00		80.25	79.48–81.01	
Wild	72.69	70.06–75.31		76.78	75.11–78.45	
ALK fusion status			0.004			0.046
Fusion	61.80	47.14–76.46		70.35	58.69–82.02	
Wild	75.87	74.30–77.45		79.30	78.42–80.19	
ROS1 fusion status			0.713			0.830
Fusion	NA	NA		NA	NA	
Wild	NA	NA		NA	NA	
Numbers of LN resected			0.488			0.297
<6	71.51	68.21–74.80		74.73	72.48–76.99	
≥6	75.67	73.89–77.46		79.29	78.30–80.27	
Stations of LN resected			0.586			0.269
<3	71.94	68.73–75.16		74.65	72.31–76.98	
≥3	75.53	73.72–77.34		79.29	78.30–80.28	
Surgical approach			0.854			0.133
Lobectomy	75.43	73.64–77.21		79.35	78.39–80.30	
Sub‐lobectomy	70.05	66.74–73.35		71.88	69.20–74.57	

**TABLE 4:** Cox regression analysis of factors associated with DFS and OS in stage IA invasive adenocarcinoma (*N* = 494)

Variable	DFS			OS		
	HR	95% CI	*p* value	HR	95% CI	*p* value
Sex: female/male	0.53	0.22–1.27	0.153	0.32	0.06–1.59	0.162
Smoking status: nonsmoker/smoker	0.98	0.42–2.30	0.968	0.82	0.20–3.26	0.773
Predominant subtype: LPA/the others	0.36	0.16–0.84	0.018	0.22	0.03–1.70	0.145
EGFR: mutant/wild	0.69	0.35–1.38	0.295	0.28	0.06–1.32	0.107
ALK: fusion/wild	2.01	0.70–6.22	0.187	2.05	0.41–10.21	0.380
Radiological type: SN/the others	3.45	1.21–9.88	0.021	218,468	0–8.71E203	0.958

### 3.4 Distribution and outcomes of EGFR mutations by radiological subtype of IAC

**FIGURE 4:**
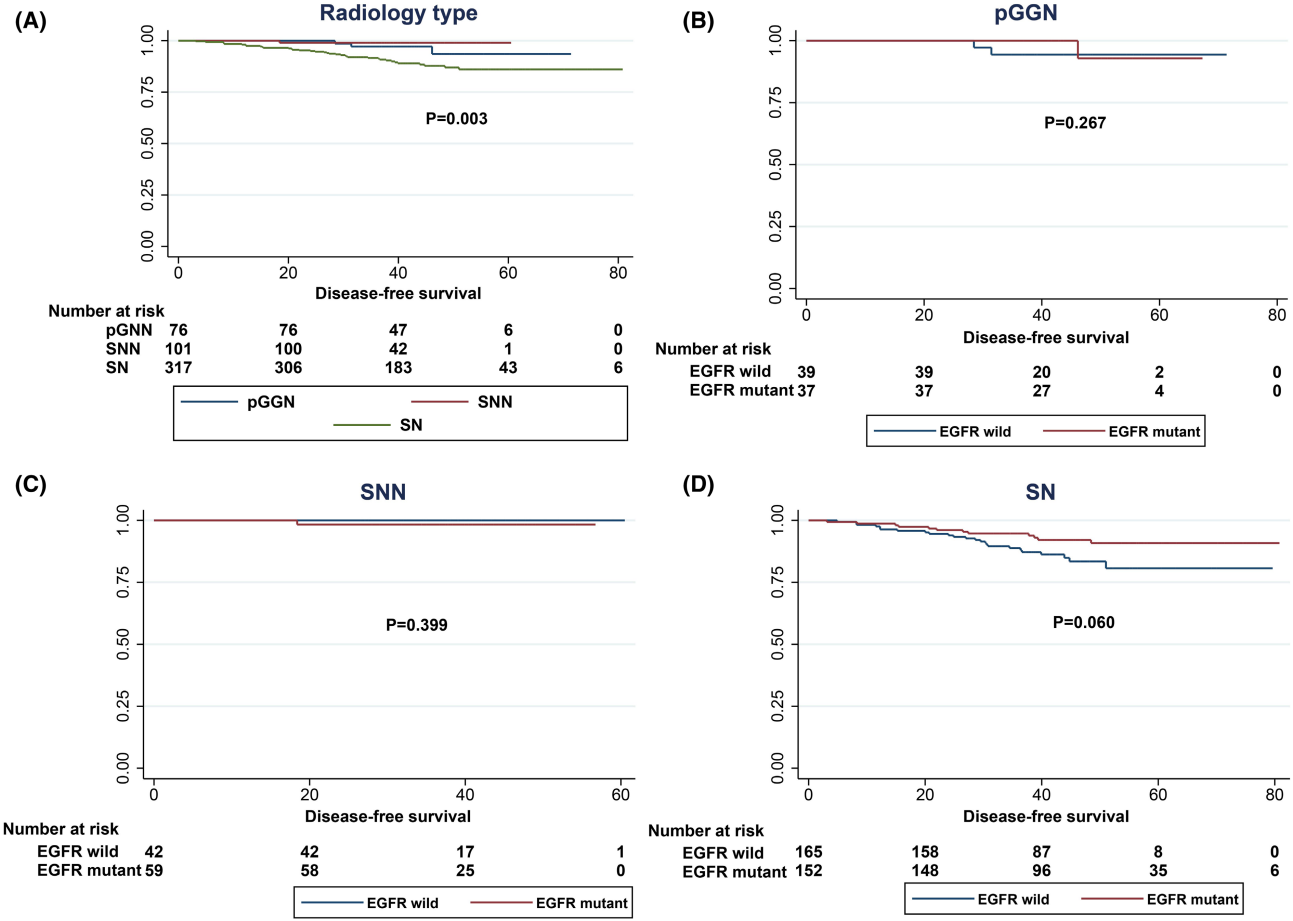
DFS curves of different EGFR mutation statuses stratified by radiological type. (A) DFS curves of all patients according to EGFR mutation status. (B) DFS curves of pGNN patients according to EGFR mutation status. (C) DFS curves of SNN patients according to EGFR mutation status. (D) DFS curves of SN patients according to EGFR mutation status.

**FIGURE 5:**
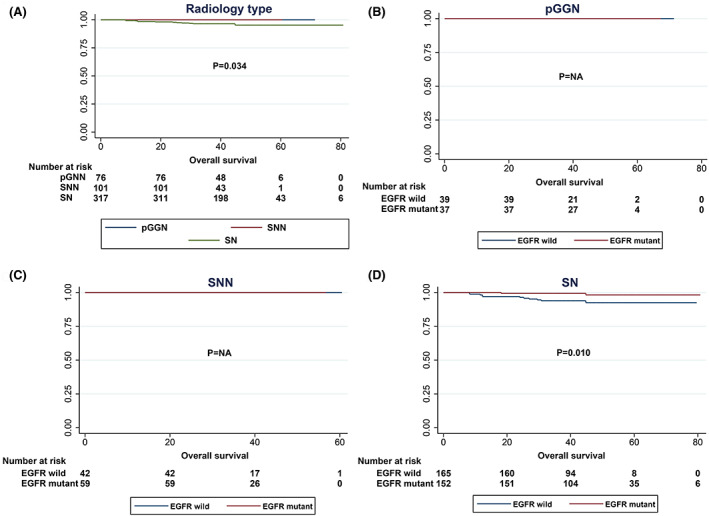
OS curves of different EGFR mutation statuses stratified by radiological type. (A) OS curves of all patients according to EGFR mutation status. (B) OS curves of pGNN patients according to EGFR mutation status. (C) OS curves of SNN patients according to EGFR mutation status. (D) OS curves of SN patients according to EGFR mutation status.

Further analysis of radiological types revealed that the mutation frequencies of EGFR mutations in pGGN, SNN and SN were 48.7%, 58.4% and 47.9%, respectively, *p* = 0.179 (Table 2). In the subgroups with radiological features of pGGN and SNN, EGFR‐mutant and EGFR‐wild‐type patients had comparable DFS (Figure 4A–C) and OS (Figure 5A–C). Notably, in the subgroup with radiological features of SN, EGFR‐mutant patients had better DFS and OS than EGFR‐wild‐type patients (Figures 4D and 5D).

### 3.5 EGFR mutation in the predominant pathological subtype of IAC

**FIGURE 6:**
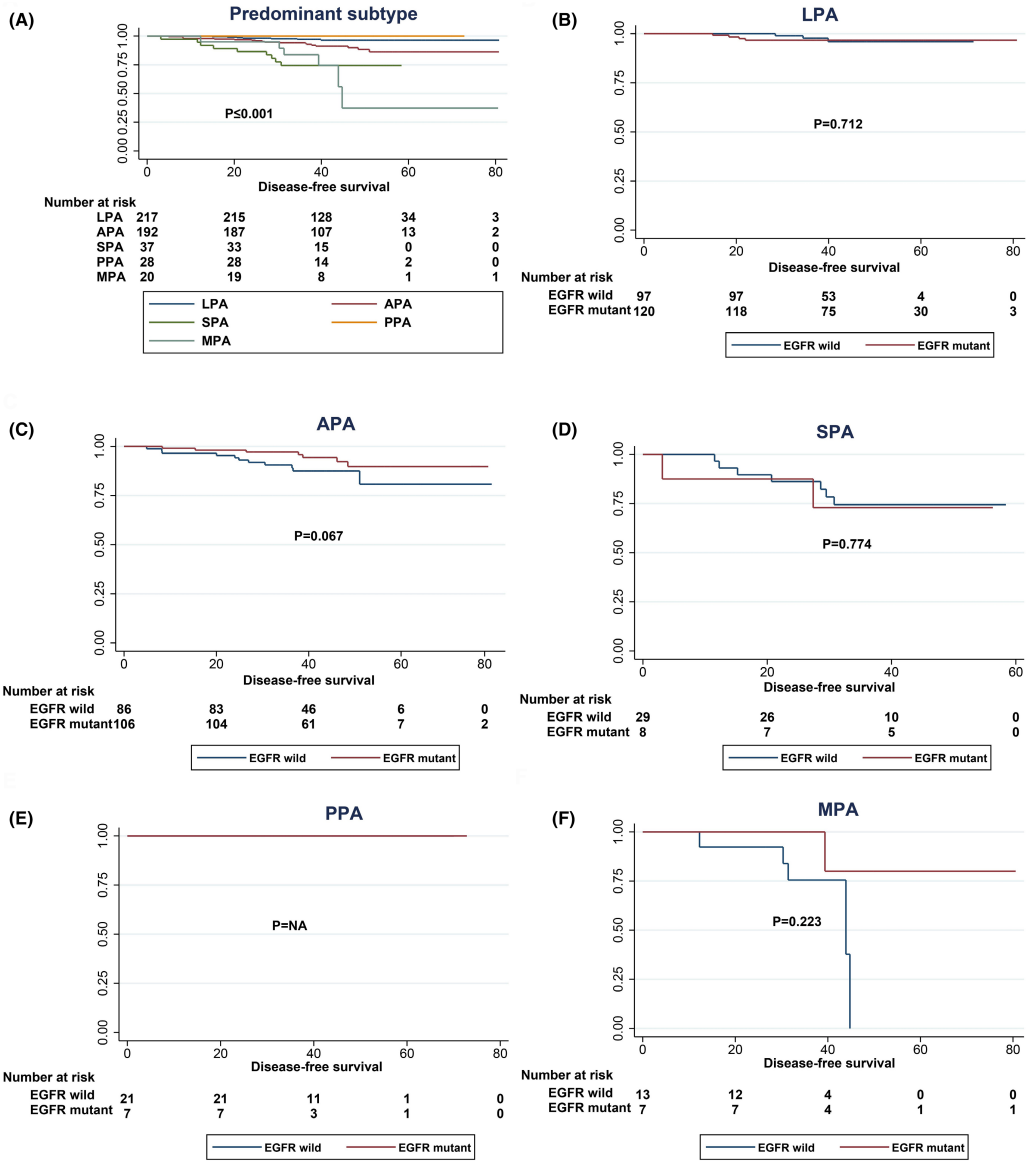
DFS curves of different EGFR mutation statuses stratified by predominant subtype. (A) DFS curves of all patients according to EGFR mutation status. (B) DFS curves of LPA patients according to EGFR mutation status. (C) DFS curves of APA patients according to EGFR mutation status. (D) DFS curves of SPA patients according to EGFR mutation status. (E) DFS curves of PPA patients according to EGFR mutation status. (F) DFS curves of MPA patients according to EGFR mutation status.

**FIGURE 7:**
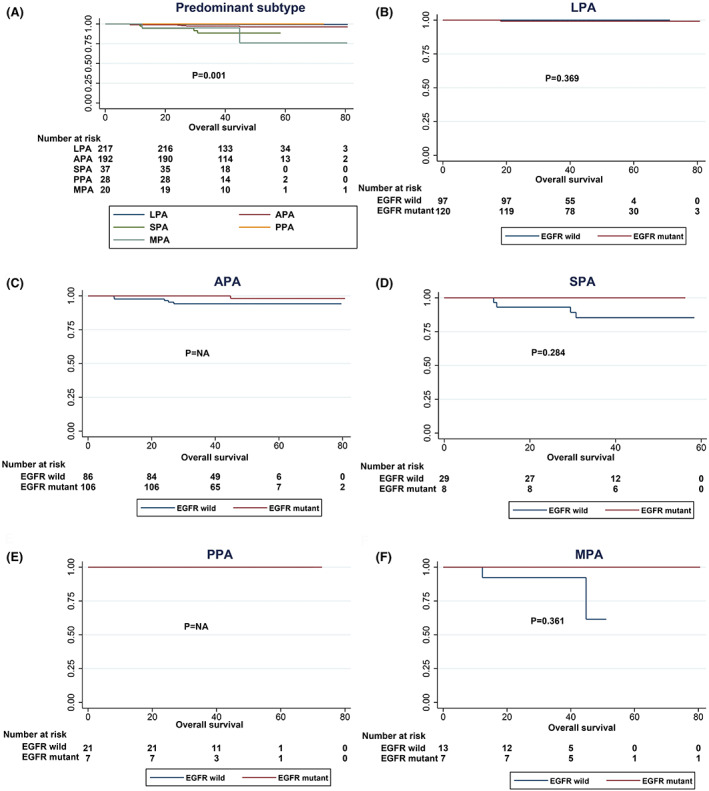
OS curves of different EGFR mutation statuses stratified by predominant subtype. (A) OS curves of all patients according to EGFR mutation status. (B) OS curves of LPA patients according to EGFR mutation status. (C) OS curves of APA patients according to EGFR mutation status. (D) OS curves of SPA patients according to EGFR mutation status. (E) OS curves of PPA patients according to EGFR mutation status. (E) OS curves of MPA patients according to EGFR mutation status.

To further assess the prognostic impact of EGFR mutations, we analysed DFS and OS analyses stratified by predominant subtype (Figures 6 and 7). Although both univariate and multivariate analyses proved that the predominant subtype is an important factor affecting DFS in patients with stage IA invasive adenocarcinoma, EGFR mutation status may have affected DFS except for that in the APA subgroup (*p* = 0.067). Negative results were obtained in the remaining subcategories (Figure 6). The same results were found in OS analysis (Figure 7), suggesting that prognostic differences between the various predominant subtypes may not be dominated by the mutation status of EGFR single genes.

## 4 DISCUSSION

For patients with stage IV non‐small‐cell lung cancer (NSCLC), the mutation status of driver genes (EGFR, ALK, ROS1, etc.) is the best predictor of the efficacy of targeted therapy. TKI therapy targeting the above driver genes has unprecedentedly turned malignant tumours into a “chronic disease”.^16^, ^17^, ^18^ In clinical practice, due to the rare recurrence and metastasis in AIS and MIA patients, the guidelines do not recommend routine driver gene detection. However, sporadic studies have demonstrated that driver gene mutations may play a significant role in the progression of LUAD from precancerous lesions to IAC.^19^, ^20^ Here, we conducted high‐volume research on the distribution of driver gene status in the three stages of AIS‐MIA‐IAC and found that the role of driver genes changes with disease progression.

The role of driver gene status in AIS or MIA is currently controversial. In the study published by Kobayashi et al.,^21^ EGFR gene detection was performed in 104 GGN specimens from 96 patients, and with the progression of AIS‐MIA‐IAC, the EGFR mutation frequency increased from 1.5% to 58.2%. Interestingly, compared with patients whose nodules were stable and unchanged for 2 years, patients with enlarged nodules had an increased probability of EGFR mutations. Several other reports also confirmed the involvement of EGFR in the malignant evolution of LUAD.^9^, ^22^ Based on the results of these studies, EGFR mutations are considered to drive the progression of LUAD from AIS to MIA. However, Zhang and colleagues^23^ reported negative results, finding that EGFR was the most frequently mutated gene. The mutation rate of EGFR in AIS was 31.3% (5/16), while that in IAC was 50% (7/14), *p* = 0.46, suggesting that EGFR mutations of AIS are comparable to those of IAC. In our study, the increased frequency of EGFR mutations may have driven the progression from AIS to MIA (33.3% vs. 50.8%, *p* = 0.004), whereas the frequency of EGFR mutations remained largely unchanged at the MIA‐to‐IAC stage (50.8% vs. 50.2%, *p* = 0.922). Likewise, neither ALK nor ROS1 gene fusions were identified in AIS. In sum, the accumulation of driver gene mutation frequency may initiate steps from preneoplasia to MIA in LUAD.

AIS/MIA, with ground‐glass nodules on imaging, these two indolent disease states are considered fully curable. Recently, a study on the 10‐year survival of AIS and MIA reported its results from a total of 524 patients who underwent radical resection for AIS (207 cases, 39.5%) and MIA (317 cases, 60.5%). Encouragingly, no disease recurrence was observed in either AIS or MIA. The 10‐year postoperative DFS rates for AIS/MIA cases were 100%/100% (*p* = 0.72), and the OS rates were 95.3%/97.8% (*p* = 0.94), respectively. Several other retrospective studies also support this view.^24^, ^25^ In the most recent study, for AIS, there were no recurrences and no deaths, and for MIA, only one patient had a lung cancer‐specific death, suggesting that although the prognosis of AIS/MIA is excellent, its follow‐up should not be ignored. Consistent with our findings, Jia and colleagues^26^ analysed the prognosis of 121 AIS/MIA patients in an Asian population with long‐term follow‐up. Among the 62 MIA cases, 2 patients developed recurrence.

To investigate the prognostic predictive role of driver gene status in invasive adenocarcinoma, we performed a survival analysis on 494 patients with stage IA IAC. It is worth noting that although no negative results were obtained by multivariate analysis, univariate analysis confirmed that EGFR mutation (protective factor, *p* = 0.036) and ALK fusion (risk factor, *p* = 0.004) were vital variables affecting the DFS of these patients. Reviewing the relevant literature, there is no conclusion on whether EGFR mutation is a prognostic predictor of operable NSCLC.^27^, ^28^, ^29^ He et al.^30^ designed a meta‐analysis of the impact of EGFR mutation status on the prognosis of operable NSCLC, including a total of 4872 patients from 19 relevant studies. Through meta‐analysis, they observed that the DFS of EGFR‐mutant patients was generally similar to that of wild‐type patients (HR 0.93, 95% CI 0.74 to 1.17). Similar results were observed in the stage I subgroup (HR 0.82, 95% CI 0.50 to 1.33). Given the influence of histological type and imaging features on patient prognosis, Deng et al.^31^ obtained a positive result; as expected, in the entire cohort, there was no difference in DFS between EGFR‐mutated and wild‐type patients (*p* = 0.266). Multivariate analysis showed that patients with EGFR mutations had solid tumours (*p* ≤ 0.001) and APA/PPA (*p* ≤ 0.001), and patients with stage II + III disease had poorer DFS independent prognostic factors (*p* = 0.004). Our study also confirmed that EGFR may be a prognostic factor in patients with SN (*p* = 0.060) and APA (*p* = 0.067), which suggested that the prognostic, predictive effect of EGFR is heterogeneous in different predominant subtypes and radiological types. This result is consistent with the abovementioned results of the OS analysis conducted by Deng et al. after excluding AIS/MIA/LPA and pGGN.

As a retrospective investigation, this study inevitably has related limitations. First, although we analysed the three most common driver genes (EGFR, ALK, and ROS1) in LUAD, results based on whole‐exome sequencing were lacking. Second, although the median follow‐up time of this study was as long as 42.57 months, long‐term follow‐up results are still lacking for the prognostic analysis of AIS and MIA. Finally, the conclusions of this study are based on large sample data from a single centre, and multicentre studies are still needed to verify the relevant conclusions.

In summary, an increased frequency of EGFR mutations dominates the progression of LUAD from AIS to MIA. For stage IA invasive adenocarcinoma, EGFR‐mutation‐positive patients may have a better prognosis, whereas patients with AIK fusions show the opposite outcome. For IAC diagnosed as stage IA, the prognostic impact of EGFR mutations may be related to the predominant subtype and radiological type subclassification.

## AUTHOR CONTRIBUTIONS

**Jianfei Zhu:** Conceptualization (lead). **Wenchen Wang:** Data curation (equal). **Yanlu Xiong:** Formal analysis (equal); methodology (equal); resources (equal); writing – original draft (equal). **Shuonan Xu:** Data curation (equal). **Jiankuan Chen:** Data curation (equal). **Miaomiao Wen:** Data curation (equal). **Yabo Zhao:** Data curation (equal). **Jie Lei:** Writing – review and editing (equal). **Tao Jiang:** Conceptualization (lead); writing – review and editing (equal).

## FUNDING INFORMATION

This study was funded by the Discipline Innovation Development Plan Project of Tangdu Hospital (2021LCYJ005), National Natural Science Foundation of China (82002421), and Special Fund for Elite Talents of Shaanxi Provincial People's Hospital (2021JY‐20).

## CONFLICT OF INTEREST

We declare that we have no conflicts of interest.

## ETHICS STATEMENT

This study was approved by the Ethics Committee of Fourth Military Medical University Affiliated Tangdu Hospital (K202107‐19), and all recruited patients signed an informed consent form.

## Data Availability

All data are shown in the manuscript and supplemental materials.
